# The role of gut microbiota–immune–endocrine crosstalk in the pathogenesis of osteoporosis

**DOI:** 10.3389/fimmu.2026.1813653

**Published:** 2026-04-22

**Authors:** Xingwen Xie, Xin Chen, Zhong Wang, Yangyang Chen, Jiawen Li

**Affiliations:** 1 Gansu University of Traditional Chinese Medicine, Lanzhou, China; 2 Affiliated Hospital of Gansu University of Traditional Chinese Medicine, Lanzhou, China

**Keywords:** dysbiosis, estrogen deficiency, gut microbiota, gut–bone axis, immune–endocrine crosstalk, lipopolysaccharide (LPS)–TLR4–NF-κB signaling, short-chain fatty acids (SCFAs), Th17/Treg imbalance

## Abstract

Osteoporosis (OP) is a common metabolic bone disorder characterized by decreased bone mass and deterioration of bone microarchitecture that result in increased bone fragility and fracture risk, especially in postmenopausal women and older adults. The gut microbiota–immune–endocrine axis has recently emerged as an important regulator of bone homeostasis, but its mechanistic role in OP pathogenesis remains incompletely understood. This review synthesizes current evidence on how gut dysbiosis, immune dysregulation, and endocrine changes interact to promote bone loss. Clinical and preclinical studies indicate that gut dysbiosis in OP is characterized by reduced microbial diversity and an increased Firmicutes/Bacteroidetes ratio, leading to altered levels of key microbial metabolites—such as decreased short-chain fatty acids (SCFAs) that normally promote bone formation, and increased lipopolysaccharide (LPS) that drives inflammation. Immune changes include chronic low-grade inflammation with elevated pro-inflammatory cytokines [e.g., tumor necrosis factor-α (TNF-α) and interleukin-6 (IL-6)] and an imbalanced T-cell profile skewed toward osteoclastogenic T helper 17 (Th17) over anti-osteoclastogenic regulatory T (Treg) cells, which together favor bone resorption. Endocrine factors further modulate this gut–bone crosstalk: estrogen deficiency (in postmenopausal OP) promotes gut dysbiosis and Th17 expansion; excess glucocorticoids compromise the gut barrier and induce dysbiosis; gut-derived incretin hormones [e.g., glucagon-like peptide-1 (GLP-1) and peptide YY (PYY)] are influenced by microbial metabolites like butyrate; and parathyroid hormone (PTH) effects on bone are both regulated by and dependent on the gut microbiota. Overall, OP can be viewed as a multi-system disorder involving an interplay among the gut microbiome, the immune system, and the endocrine system. This integrated perspective on the “gut–bone axis” suggests that interventions targeting the gut microbiota (probiotics, prebiotics, etc.) could complement traditional therapies for OP. Enhancing skeletal health may require a multidisciplinary approach that considers gut microbial status, immune function, and hormonal milieu in tandem.

## 1 Introduction

Osteoporosis (OP) is the most prevalent metabolic bone disease, defined by low bone mass and microarchitectural deterioration of bone tissue that lead to enhanced bone fragility and fracture susceptibility (1, 2). OP affects hundreds of millions of people worldwide—predominantly postmenopausal women and older men—and has become a major public health concern (3). According to the International Osteoporosis Foundation, over 200 million people globally have OP, and one osteoporotic fracture occurs every 3 s (4, 5). Approximately half of women above 50 will experience an osteoporotic fracture, largely due to postmenopausal osteoporosis (PMO) precipitated by estrogen deficiency and accelerated bone loss (6, 7). The insidious onset and substantial disability burden of OP have led to it being called a “silent epidemic.” The clinical and socioeconomic impact of OP continues to grow as populations age (8).

The pathogenesis of OP involves an imbalance in bone remodeling, whereby bone resorption by osteoclasts outpaces bone formation by osteoblasts (9). Traditionally, research has focused on endocrine regulators [e.g., sex steroids and parathyroid hormone (PTH)] and local signaling pathways (e.g., the RANKL/RANK/OPG axis) that govern bone turnover. However, emerging evidence supports a conceptual bone–microbial–immune–endocrine network, highlighting the gut microbiota as a key regulator of immune homeostasis and endocrine signaling, which, through the gut–bone axis, influences OP development and progression (10–12). The gut microbiota is a vast microbial ecosystem in the gastrointestinal tract that co-evolves with the host and contributes to nutrient metabolism, mucosal barrier function, and immune maturation (13, 14). Growing evidence links gut dysbiosis to metabolic bone disease, giving rise to the concept of a gut–bone axis (a facet of osteoimmunology) (15–17). Increasingly, the gut, immune, and endocrine systems are seen as a highly interactive and interrelated network critical for skeletal homeostasis (18–20). Indeed, the gut harbors a large proportion of the body’s immune cells and plays a central role in immune regulation, while the immune system in turn significantly influences bone remodeling. Disruptions in gut microbial homeostasis can thus provoke immune imbalances that adversely affect bone mass. This interconnected perspective underscores the importance of integrating gut, immune, and bone health when considering OP pathogenesis.

## 2 Mechanistic links between the gut microbiota and bone metabolism

### 2.1 Gut dysbiosis and osteoporosis

Patients with OP often exhibit gut dysbiosis. The gut microbial diversity in individuals with OP is significantly lower than that in healthy controls (21, 22). Typical findings include an increased Firmicutes-to-Bacteroidetes (F/B) ratio, higher relative abundance of certain genera (e.g., *Bacteroides*, *Clostridium*, and *Lactobacillus*), and a marked reduction in short-chain fatty acid (SCFA)-producing commensals (e.g., *Ruminococcus*) (23, 24). Similar compositional shifts were observed in older Chinese cohorts, where OP was associated with increases in *Bacteroides* and *Eisenbergiella* and decreases in *Ruminococcus* (16, 23). Such dysbiosis is thought to disrupt skeletal homeostasis: expansion of pro-inflammatory or opportunistic taxa can enhance host inflammation, reduce nutrient absorption, and trigger osteoclastogenic signaling, accelerating bone degradation (25, 26). For example, overgrowth of potentially pathogenic bacteria like Parabacteroides and Eggerthella can increase the release of pro-inflammatory molecules (endotoxins), while loss of beneficial microbes like *Bifidobacterium* impairs anti-inflammatory regulation in the gut (27, 28).

### 2.2 Microbial metabolites as remote regulators of bone

The gut microbiota exerts remote control over bone metabolism via a variety of metabolites (29). Two of the most studied mediators are SCFAs and lipopolysaccharide (LPS). Fermentation of dietary fiber by gut microbes produces SCFAs (acetate, propionate, and butyrate), which generally enhance bone formation and protect against bone loss (30, 31). Butyrate, in particular, promotes osteoblast differentiation and mineralization, increasing bone density in experimental models (32). Mechanistically, butyrate upregulates Wnt signaling in bone marrow stromal cells, promoting their osteogenic differentiation (33). Additionally, butyrate expands FoxP3^+^ regulatory T (Treg) cells and suppresses pro-inflammatory T helper 17 (Th17) cell differentiation (via G-protein-coupled receptors like GPR43 on immune cells), creating an immune-tolerant environment that indirectly inhibits osteoclast development. These immune-mediated effects complement butyrate’s direct actions on osteoblasts to inhibit bone resorption (34). Consistent with these findings, butyrate supplementation or enrichment of butyrate-producing bacteria (e.g., *Lactobacillus rhamnosus* GG) has demonstrated significant increases in trabecular bone mass in animal studies (35, 36).

By contrast, LPS—an endotoxin from Gram-negative bacteria—can enter circulation when the intestinal barrier is compromised, triggering systemic inflammation. The effects of LPS on bone are dose- and context-dependent: at low concentrations *in vitro*, LPS can induce osteoclast precursors to adopt macrophage-like properties in the absence of RANKL, or promote osteoclastogenesis via TLR4 signaling when RANKL is present (37). *In vivo*, elevated gut permeability permits LPS translocation into the bloodstream, leading to systemic immune activation and accelerated bone loss (1). Mechanistically, LPS binds TLR4 on innate immune cells (e.g., monocytes/macrophages), activating NF-κB and inducing production of pro-inflammatory cytokines [tumor necrosis factor-α (TNF-α), interleukin-6 (IL-6), and interleukin-1β (IL-1β)] that drive osteoclastogenesis and bone resorption. In general, SCFAs (especially butyrate) are bone-protective—suppressing osteoclasts and stimulating osteoblasts—whereas endotoxins like LPS promote bone loss via inflammatory pathways (1). These metabolites serve as key “messengers” of the gut–bone axis, transmitting signals from the gut environment to skeletal tissue (38).

Beyond SCFAs and LPS, many other microbial metabolites influence bone. Secondary bile acids (produced by gut microbial conversion of primary bile acids) can modulate skeletal cells by activating various nuclear receptors (39). Some secondary bile acids (e.g., isoalloLCA) preferentially expand Treg cells, conferring immunomodulatory and anti-osteoporotic effects (40, 41), whereas others affect Th17 differentiation through specific receptors (40). Tryptophan metabolites (e.g., indoles) act via the aryl hydrocarbon receptor (AhR) on immune and stromal cells and have been implicated in microbiota-dependent regulation of bone mass (41). Additionally, microbially produced vitamins (e.g., vitamin K_2_) and polyamines support skeletal health (42). For example, vitamin K_2_ from commensals promotes carboxylation of osteocalcin and other bone matrix proteins, enhancing bone mineralization (43). Gut microbes may also lower intestinal pH through organic acid production, improving mineral solubility and absorption and helping preserve bone mineral density (44, 45).

**Figure 1:**
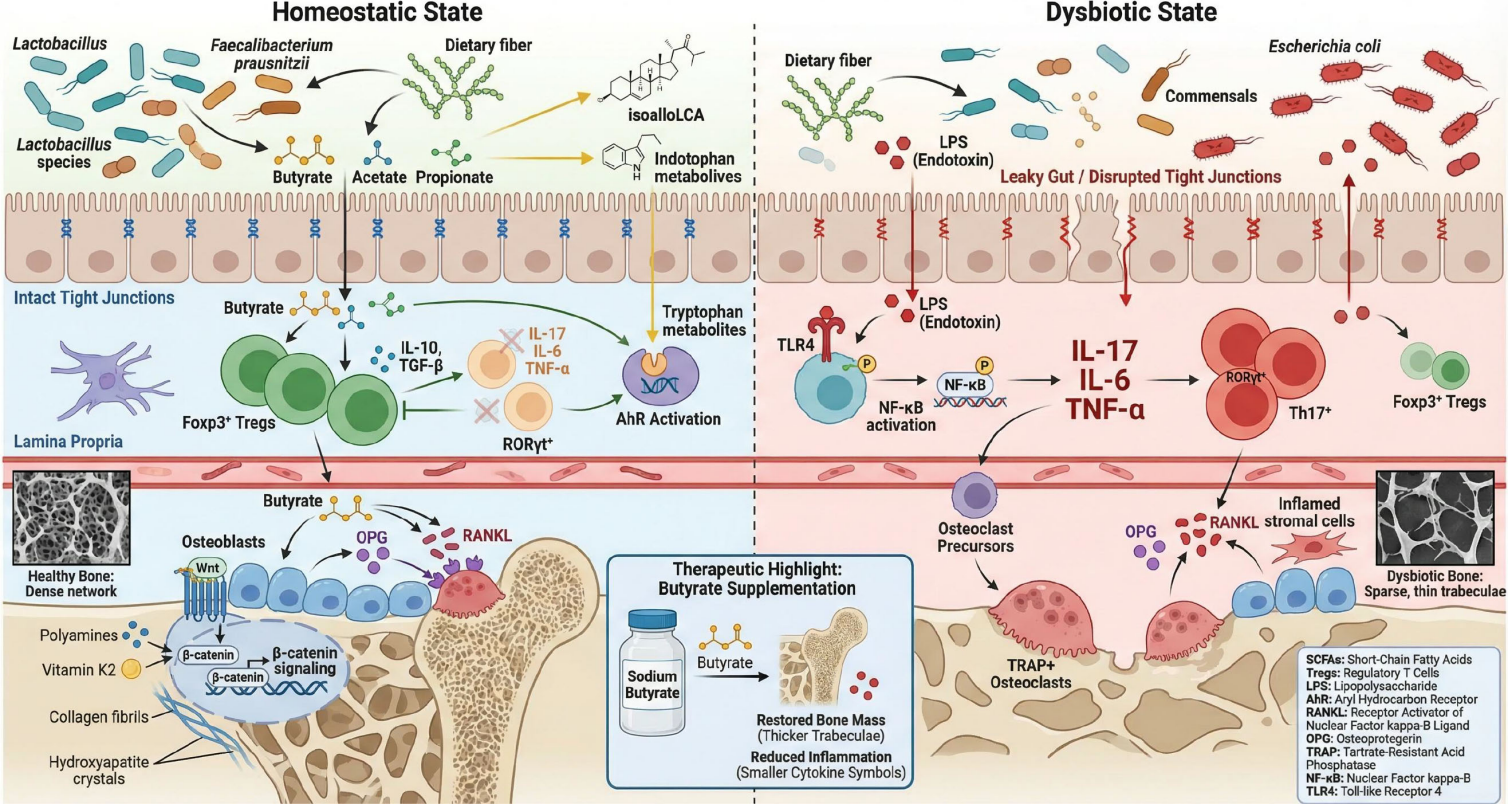
Schematic representation of gut microbiota–immune–bone crosstalk in homeostatic vs. dysbiotic states. During homeostasis, dietary fiber is fermented by commensal bacteria (e.g., Lactobacillus and butyrate producers) to generate SCFAs (acetate, propionate, butyrate). SCFAs induce expansion and stabilization of Treg cells, which inhibit Th17 cell polarization and reduce pro-inflammatory cytokine production (IL-17, IL-6, TNF-α). Butyrate also indirectly suppresses osteoclastogenesis by modulating the RANKL/OPG ratio, and it promotes osteoblast differentiation via Wnt/β-catenin signaling. Microbiota-derived secondary bile acids (e.g., isoalloLCA) further promote Treg differentiation, and microbial tryptophan metabolites activate AhR pathways to maintain immune tolerance. Microbial vitamins and polyamines additionally support immune homeostasis and bone mineralization. During dysbiosis, loss of beneficial microbes and overgrowth of harmful species (“leaky gut”) increase intestinal permeability, allowing microbial products (e.g., LPS) to translocate into circulation. LPS triggers TLR4/NF-κB–mediated inflammatory cascades and systemic cytokine release. Heightened inflammation accelerates osteoclast precursor recruitment and RANKL-driven osteoclastogenesis, leading to excessive bone resorption. An imbalance between Treg-mediated tolerance and Th17-driven inflammation disrupts skeletal homeostasis, contributing to osteoporotic bone loss. Notably, experimental studies show that modulating the gut–bone immune axis (e.g., via butyrate supplementation) increases trabecular bone mass, highlighting the therapeutic potential of this pathway.

### 2.3 Intestinal barrier dysfunction and inflammation

Not all microbiota influences on bone are mediated by metabolites; intestinal barrier integrity and immune signaling are also key factors (46). Under normal conditions, pathogenic microbes and endotoxins are confined to the gut lumen and do not elicit systemic inflammation. However, in dysbiosis accompanied by barrier disruption, bacterial products like LPS more readily breach the epithelium and enter circulation, provoking chronic low-grade inflammation (47). This systemic inflammatory state drives monocyte–macrophage lineage cells to differentiate into osteoclasts and enhances bone resorption, largely via pro-inflammatory cytokines such as TNF-α and IL-6 (48, 49). Clinical and animal studies likewise link gut barrier dysfunction to higher OP risk: for instance, high-fat diet-induced obesity, chronic psychological stress, or endotoxemia-related bone loss are associated with reduced tight junction proteins and increased intestinal permeability. Conversely, interventions that preserve or restore barrier integrity (e.g., mucosal protectants) have been shown to alleviate inflammation-induced bone loss.

## 3 Immune mechanisms: microbiota-mediated osteoimmune regulation

The gut microbiota is tightly integrated with the host immune system, together shaping the osteoimmune network. Dysbiosis disrupts immune equilibrium and influences bone remodeling through multiple pathways (28). Below, we summarize how microbiota–immune interactions contribute to OP, focusing on inflammatory cytokines and T-cell subsets.

### 3.1 The inflammatory milieu in the skeletal system

Chronic low-grade inflammation is a key driver of OP. Gut dysbiosis is often accompanied by elevated systemic levels of pro-inflammatory cytokines, including TNF-α, IL-1β, and IL-6 (50). These mediators, produced in abundance by activated macrophages and T cells, disrupt bone homeostasis (51). TNF-α and IL-1β not only directly promote osteoclast differentiation and activity but also upregulate RANKL expression, amplifying bone resorption (52, 53). Consistent with this paradigm, germ-free animals—lacking microbial stimulation—exhibit markedly lower levels of pro-inflammatory mediators and fewer osteoclasts (54). Germ-free mice have significantly reduced expression of RANKL, TNF-α, and IL-6 in bone, resulting in fewer osteoclasts and, paradoxically, increased bone mass (55). Colonization of germ-free mice with a normal microbiota restores pro-inflammatory cytokine production and osteoclast numbers toward baseline. These findings suggest that a baseline level of microbial stimulation (and corresponding immune activation) is required for normal bone remodeling, whereas dysbiosis-induced excessive inflammation skews remodeling toward pathological bone resorption (56, 57). Clinically, chronic inflammatory diseases like rheumatoid arthritis and inflammatory bowel disease are frequently complicated by secondary OP, sharing the hallmark of elevated systemic cytokines (58). Accordingly, strategies that curb chronic inflammation—potentially by modulating the gut microbiota—may help reduce OP risk (59).

### 3.2 T-cell subsets: imbalance between Th17 and Treg cells

Th17 and Treg cells are key players in osteoimmunology (60). Th17 cells, a subset of effector CD4^+^ T cells, secrete IL-17 and are strongly pro-osteoclastogenic (61, 62). IL-17 stimulates osteoblastic stromal cells to upregulate RANKL and chemokines, hastening osteoclast differentiation and recruiting osteoclast precursors to bone (18, 63). In the context of estrogen deficiency, Th17 cells expand and become major drivers of bone loss in PMO (18). Indeed, serum IL-17A levels in postmenopausal women negatively correlate with bone mineral density, and high IL-17 levels are associated with increased osteoclast activity and inhibited osteoblast function (64).

Treg cells, conversely, are anti-inflammatory CD4^+^ T cells that produce IL-10 and transforming growth factor-β (TGF-β), which inhibit inflammatory responses and block osteoclast formation (64). Treg cells can also suppress osteoclast precursors via direct cell–cell contact, using immune checkpoint molecules like CTLA-4, thereby protecting bone during remodeling (17). Under homeostatic conditions, Th17 and Treg cells exist in a dynamic equilibrium that can be disturbed by dysbiosis or immune dysregulation (62). Certain gut bacteria [e.g., segmented filamentous bacteria (SFB)] potently stimulate Th17 differentiation and can drive excessive Th17 expansion in the gut and bone marrow (17). On the other hand, microbial metabolites like butyrate and specific secondary bile acids (e.g., isoalloLCA) promote Treg proliferation and function, thereby restraining inappropriate bone resorption (18). Notably, mice colonized with SFB exhibit significant trabecular bone loss accompanied by increased IL-17^+^ TNF-α^+^ Th17 cells in bone marrow (17). Butyrate supplementation, by activating GPR43 on dendritic cells, induces Treg cells and in turn stimulates bone marrow CD8^+^ T cells to express the osteogenic factor Wnt10b, promoting bone formation (18, 23). Together, microbiota-driven shifts in the Th17/Treg balance reorganize the osteoimmune environment: a Th17-dominant response promotes OP, whereas a Treg-dominant response protects bone (61, 63).

**Figure 2:**
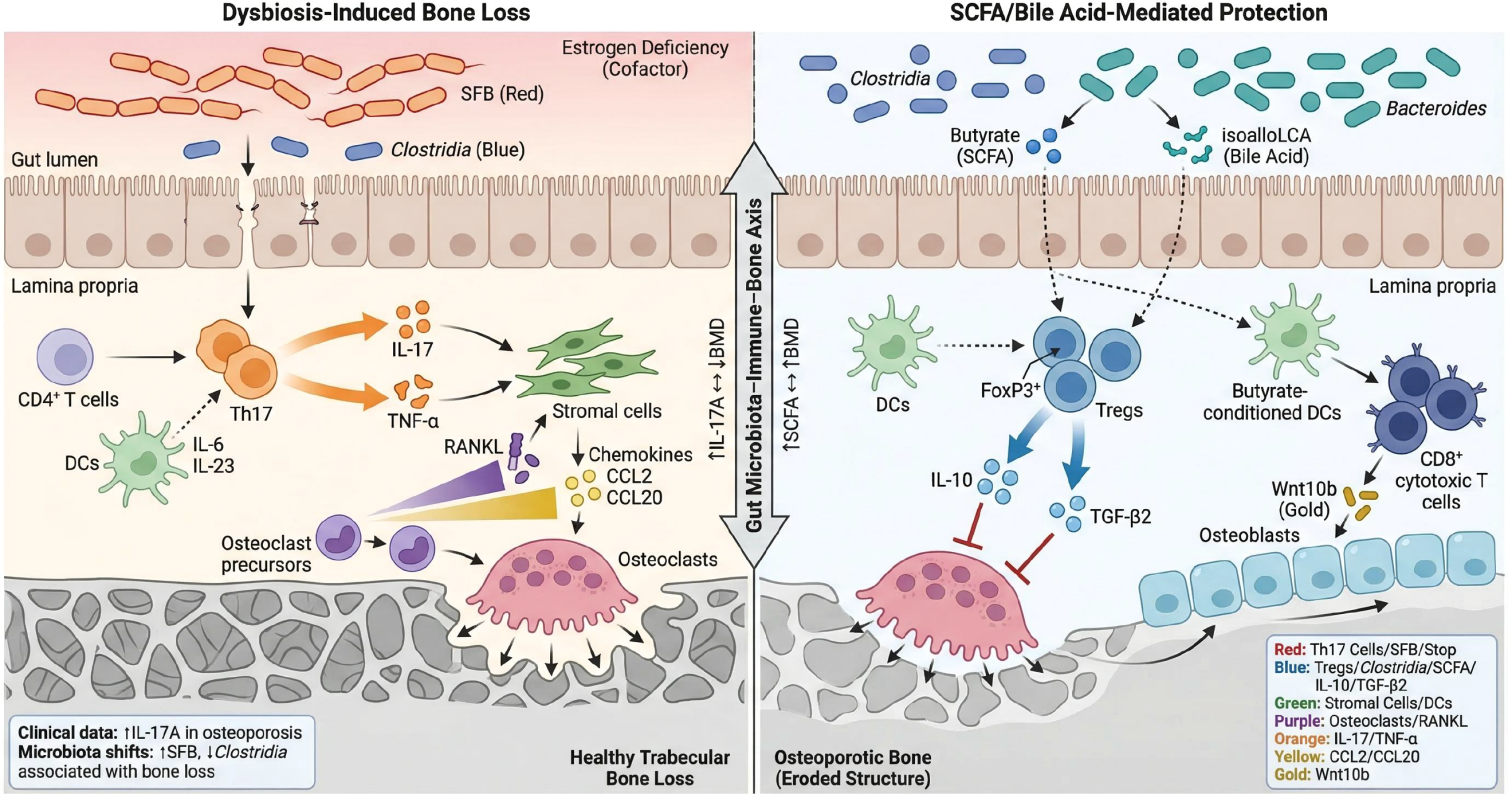
Gut microbiota-driven immune modulation of bone remodeling via Th17/Treg balance. Microbial dysbiosis (e.g., SFB overgrowth, loss of SCFA-producing commensals) skews T-cell differentiation toward osteoclastogenic Th17 cells, especially under estrogen-deficient conditions. Th17 cells secrete IL-17 and TNF-α, which stimulate stromal and osteoblastic cells to upregulate RANKL and chemokines (CCL2, CCL20), promoting osteoclast precursor recruitment and differentiation. The outcome is near-complete loss of trabecular bone and a Th17-dominant osteoporotic phenotype. Conversely, microbiota-derived metabolites like butyrate (SCFAs) and isoalloLCA (bile acid) favor Treg activation and homeostasis. Treg cells inhibit osteoclast proliferation by secreting IL-10, TGF-β2 and via cell contact mechanisms (CTLA-4, LAG-3). Meanwhile, butyrate-conditioned dendritic cells (through GPR43) induce CD8⁺ T cells to produce Wnt10b, enhancing osteoblast activity and bone formation. In summary, the gut microbiota–immune–bone axis is a fundamental regulatory system for bone homeostasis. Dysbiosis-mediated Th17/Treg imbalance links microbial and hormonal changes to osteoporosis pathogenesis, as supported by clinical correlations (e.g., high IL-17A levels with low BMD) and histological evidence of trabecular bone loss.

### 3.3 Regulation of additional immune mediators

The gut microbiota also influences bone through other immune pathways. Dysbiosis often leads to heightened macrophage activation, with excessive secretion of osteoclastogenic cytokines like TNF-α and IL-6 (64). In a mouse model of ulcerative colitis, intestinal inflammation dramatically increased bone marrow levels of chemokines (G-CSF, MCP-1, and CCL5), driving expansion and migration of osteoclast precursors and disrupting remodeling balance (65, 66). Activated B cells can also produce RANKL; in microbiota-driven chronic inflammation, B cell-derived RANKL further contributes to osteoclast formation. Conversely, some microbial metabolites have anti-inflammatory effects that restrain excessive immune activation. For instance, secondary bile acids from beneficial *Clostridium* species can suppress the macrophage NLRP3 inflammasome, reducing IL-1β and other pro-resorptive mediators (67). Thus, microbial products can act as either “accelerators” or “brakes” in osteoimmunity. Host factors (age, sex, genetics, and nutrition) further shape these interactions (23). Aging, for example, is associated with chronic low-grade inflammation (“inflammaging”) and reduced microbial diversity; together, these changes likely contribute to the high OP susceptibility of the elderly (68, 69). In summary, the gut microbiota profoundly affects bone metabolism via immune modulation: a stable microbiota supports balanced immune activation and regulation, whereas dysbiosis promotes excessive pro-inflammatory mediators and Th17/Treg imbalance, ultimately leading to bone loss (60, 61). The major immune cell populations influenced by the gut microbiota and their corresponding effects on bone remodeling are summarized in **Table 1**. In addition, the major gut microbiota-derived metabolites/components and their immune-mediated effects on bone are summarized in **Table 2**.

**Table 1:** | Interactions between gut microbiota and immune cells relevant to bone remodeling.

Immune cell	Microbial influence	Effect on bone
Th17 cells (CD4^+^)	Expanded by dysbiosis (e.g., SFB overgrowth); ↑ IL-17, TNF-α (pro-inflammatory)	↑ Osteoclastogenesis (bone resorption ↑)—promotes bone loss
Treg cells (CD4^+^)	Expanded by SCFAs (butyrate), secondary bile acids; ↑ IL-10, TGF-β (anti-inflammatory)	↓ Osteoclastogenesis (bone resorption ↓)—protects bone mass
Macrophages (M1)	Activated by LPS via TLR4; secrete TNF-α, IL-6, and IL-1β (inflammation)	↑ Osteoclast formation and activity—bone loss
B cells	Dysbiosis/inflammation → B cells express RANKL	↑ Osteoclastogenesis (via RANKL)—bone loss
Dendritic cells	Butyrate via GPR43 → tolerogenic DCs (promote Treg cells); also stimulate CD8^+^ T cells (Wnt10b)	↑ Osteoblast activity (via Wnt10b); indirect ↓ osteoclasts—supports bone formation

“↑” indicates an increase or upregulation, whereas “↓” indicates a decrease or downregulation.

**Table 2:** | Key gut microbiota-derived metabolites/components and their immune-mediated effects on bone.

Microbial metabolite	Immune pathway/target	Net effect on bone
Short-chain fatty acids (SCFAs) (e.g., butyrate)	Activate GPR43 on dendritic cells → expand Treg cells; inhibit Th17; ↑ anti-inflammatory cytokines (IL-10, etc.)	Suppress osteoclastogenesis, promote osteoblast activity—bone-protective (↑ bone mass)
Lipopolysaccharide (LPS)	Binds TLR4 on monocytes/macrophages → NF-κB activation; ↑ TNF-α, IL-6 (inflammation)	Stimulates osteoclastogenesis—bone-destructive (↑ bone loss)
Secondary bile acids (e.g., isoalloLCA)	Expand Treg cells; suppress macrophage inflammasome (↓ IL-1β)	Restrain excessive bone resorption—protective
Tryptophan metabolites (e.g., indoles)	Activate AhR on immune/stromal cells; modulate Th17/Treg balance	Generally anti-inflammatory—protective (context-dependent)
Microbial vitamins (e.g., K_2_) and polyamines	Support immune homeostasis; vitamin K_2_ aids osteocalcin activation (bone matrix quality)	Enhance bone mineralization—supportive

“↑” indicates an increase or upregulation, whereas “↓” indicates a decrease or downregulation.

## 4 Endocrine mechanisms: bidirectional interactions between hormones and the gut microbiota

Endocrine regulation is central to OP, and multiple bidirectional links exist between the gut microbiota and host hormones. Hormonal changes can reshape the microbiota, and microbial dynamics can, in turn, influence hormonal activity in bone metabolism (70). Below, we discuss major hormones implicated in OP—estrogen, glucocorticoids (GCs), gut-derived incretins, and PTH—and their mechanistic interactions with the gut microbiota (71).

### 4.1 Estrogen–microbiota crosstalk

#### Dual effects of estrogen on bone and the microbiota

Estrogen (particularly 17β-estradiol) is crucial for maintaining bone mass. It directly stimulates osteoblast activity, inhibits osteoclast formation, and also exerts skeletal protection via immune modulation (68). Estrogen tightly regulates immune cells: under normal conditions, estrogen–ERα signaling in T and B cells inhibits their expression of RANKL while promoting osteoblast production of osteoprotegerin (OPG), thereby suppressing osteoclastogenesis (72). Estrogen also increases anti-inflammatory cytokines (e.g., IL-10) and reduces inflammatory signaling in the bone marrow microenvironment (68). Consequently, sufficient estrogen fosters a balanced coupling of bone formation and resorption (72).

#### Estrogen deficiency and bone loss

The sharp decline in estrogen after menopause upsets this balance, predisposing individuals to OP. Mechanistically, estrogen deficiency removes ER-mediated osteoprotection and, importantly, heightens immune-driven osteoclastogenesis (14). In estrogen-deficient conditions, T cells become overactive and secrete more TNF-α; Th17 cell fractions expand, and IL-17 from Th17 cells induces RANKL expression by bone marrow stromal cells, driving robust osteoclast proliferation (73). In mice, T cell or TNF-α ablation markedly attenuates ovariectomy (OVX)-induced bone loss, underscoring the pivotal role of immunity in estrogen deficiency OP (14). Notably, the gut microbiota is a critical intermediary. Pacifici and colleagues showed that broad-spectrum antibiotics or germ-free housing largely abrogates OVX-induced bone loss, suggesting that estrogen deficiency requires microbiota-driven immune activation to fully manifest its bone-resorptive effects. In the absence of a microbiota, estrogen deficiency fails to elicit the usual inflammatory responses or Th17 expansion, thus blunting osteoclastogenic pathways (71). Conversely, fecal microbiota transplantation (FMT) or probiotic colonization significantly ameliorates the OVX bone-loss phenotype (73, 74). Thus, postmenopausal bone loss is strongly dependent on the gut microbiota–immune axis.

#### Estrogen-mediated remodeling of the gut microbiota

Conversely, estrogen levels also feedback to modulate the gut microbiota composition and function (75). In OVX rats on high-fat diets, estradiol reintroduction increased the abundance of beneficial genera (*Lactobacillus* and *Bifidobacterium*), upregulated tight junction proteins, and improved gut barrier integrity (62). Estrogen suppresses the bloom of endotoxin-producing pathobionts (e.g., phylum Proteobacteria) while promoting mucin-degrading beneficial taxa such as *Akkermansia*, thereby supporting the mucosal barrier (76). Moreover, estrogen signaling via ERβ and GPR30 activates pathways (e.g., PI3K/Akt/mTOR) that alter microbial diversity and metabolism (77). These observations indicate that estrogen loss reshapes the gut ecosystem: estrogen deficiency is commonly followed by reduced microbial diversity, depletion of protective commensals, and overgrowth of potentially harmful taxa (75). For example, Yu et al. found that OVX prompted SFB overgrowth in mice, accompanied by increases in TNF^+^ and Th17 cells in the gut and reduced bone density (63). OVX-induced microbiota changes can also elevate bone marrow cytokines (IL-7 and IL-15), sustaining low-grade inflammation and bone loss (60). Estrogen deficiency may further increase gut permeability: Guan et al. demonstrated that loss of estrogen impairs the intestinal barrier and elevates plasma LPS, activating TLR4/NF-κB signaling and osteoclast activity (77).

The gut microbiota is also involved in estrogen’s enterohepatic circulation. Some gut bacteria produce β-glucuronidase, which deconjugates hepatic estrogen metabolites, freeing estrogen for reabsorption—a microbial function termed the estrobolome (78). Dysbiosis (e.g., reduced β-glucuronidase producers) can diminish estrogen recycling and exacerbate systemic estrogen deficiency (79). In summary, estrogen–microbiota–immune interactions form a key mechanism in PMO. Estrogen deficiency initiates dysbiosis and barrier impairment, which mobilize immune mediators (e.g., Th17 expansion) that drive osteoclastogenesis; conversely, maintaining a healthy microbiota or intervening (with probiotics or estrogen therapy) can slow this pathological process (60, 63).

**Figure 3:**
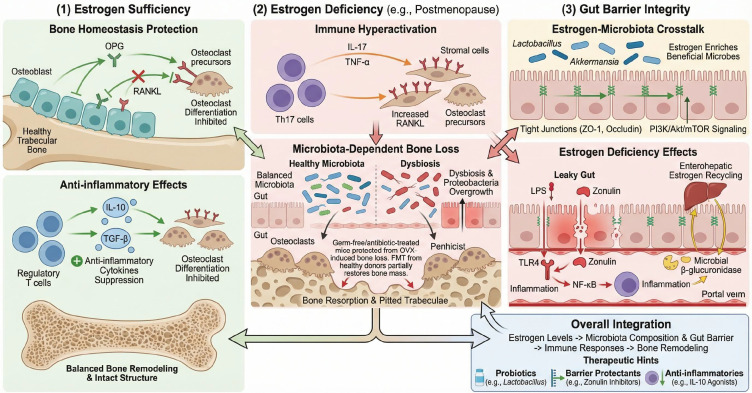
Bidirectional interactions between estrogen and the gut microbiota–immune axis in bone remodeling. (1) Estrogen sufficiency: Estrogen exerts protective effects on bone homeostasis. Estrogen–ERα signaling in osteoblasts upregulates OPG while downregulating RANKL, raising the OPG/RANKL ratio and suppressing osteoclast differentiation. Estrogen also restrains pro-inflammatory T cell activity and boosts anti-inflammatory cytokine production (e.g., IL-10), thereby preserving skeletal integrity. (2) Estrogen deficiency: Loss of estrogen (e.g., postmenopause) leads to immune hyperactivation (excess TNF-α) and an expansion of Th17 cells, causing elevated IL-17 and increased RANKL expression by stromal cells. This pro-inflammatory milieu accelerates osteoclastogenesis and bone resorption. Notably, these effects are microbiota-dependent: germ-free or antibiotic-treated animals are protected from OVX-induced bone loss, whereas dysbiotic microbiotas (e.g., Proteobacteria overgrowth) exacerbate bone loss. Fecal microbiota transplantation from healthy donors can partially restore bone mass in estrogen-deficient hosts. (3) Gut barrier effects: Estrogen–microbiota crosstalk also influences intestinal integrity. Estrogen supplementation remodels the microbiota (increasing Lactobacillus, Akkermansia; reducing Proteobacteria) and maintains tight junctions (via PI3K/Akt/mTOR signaling), preserving the mucosal barrier. Conversely, estrogen lack compromises barrier integrity, elevates zonulin-mediated permeability and allows LPS translocation, which triggers TLR4/NF-κB signaling and systemic inflammation. Microbial β-glucuronidase regulates enterohepatic estrogen recycling, linking gut metabolism to systemic estrogen levels. Overall, (1)–(3) illustrate an integrated endocrine–immune–microbial network driving bone loss in estrogen deficiency, and suggest potential therapies (e.g., probiotics, barrier protectants, anti-inflammatories) to counter postmenopausal osteoporosis.

### 4.2 Glucocorticoids and the gut–bone axis

GCs like prednisolone are widely used to treat inflammatory and autoimmune diseases, but chronic GC use leads to glucocorticoid-induced osteoporosis (GIOP). Excess GCs adversely affect bone through multiple mechanisms: they induce osteoblast and osteocyte apoptosis, inhibit osteoblast differentiation, and prolong osteoclast lifespan, collectively causing suppressed bone formation and enhanced bone resorption (80). Indirectly, GCs disrupt systemic metabolism and hormonal homeostasis (e.g., reducing sex steroid levels and altering calcium/phosphate balance), further exacerbating bone loss (80). Emerging evidence indicates that GC-induced alterations in the gut microbiota and intestinal barrier function are pivotal intermediaries of GIOP (81). In animal models, chronic GC exposure reduces gut microbial diversity and depletes beneficial taxa (82), while also damaging epithelial tight junctions and increasing gut permeability (81). These changes lead to elevated circulating LPS, persistent inflammation, and impaired bone cell activity (81). In an elegant study, Schepper et al. implanted sustained-release prednisolone pellets in mice and observed significant trabecular bone loss within 8 weeks, along with increased serum LPS; notably, administering probiotics (*Lactobacillus reuteri*) or high-molecular-weight polymers preserved barrier integrity and largely prevented bone loss (81). Interestingly, depleting the microbiota with antibiotics protected mice from GC-induced trabecular bone loss, indicating that the presence of gut microbes is a prerequisite for the full manifestation of GIOP (81). Mechanistically, microbiota depletion prevented GC-induced dysbiosis and barrier damage, thereby reducing LPS translocation and other pro-resorptive signals upstream (81). Antibiotics also blocked GC-induced upregulation of osteoblast apoptosis genes (e.g., Bax/Bcl-2 ratio) (81). These findings highlight that a substantial component of GC-induced skeletal damage is mediated via dysbiosis and secondary inflammation (81). Accordingly, gut-targeted strategies (e.g., probiotics, interventions to strengthen the gut barrier) are being explored as novel approaches to prevent or mitigate GIOP (82, 83). Early clinical data suggest that certain probiotics may improve bone turnover markers and increase bone mineral density in patients on long-term GC therapy (84). Overall, GCs affect bone through intertwined immune–microbial pathways in which disruption of microbiota homeostasis and intestinal inflammation are key drivers of bone loss; protecting the gut–bone axis may thus alleviate GC-induced skeletal side effects.

### 4.3 Gut-derived hormones (incretins) and bone

#### 4.3.1 Incretins and bone metabolism

Beyond its digestive functions, the gut acts as a major endocrine organ. Hormones secreted by intestinal L cells—collectively known as incretins—participate in bone remodeling (85). The best-studied incretins include glucagon-like peptide-1 (GLP-1), GLP-2, glucose-dependent insulinotropic polypeptide (GIP), and peptide YY (PYY) (85). Though primarily recognized for roles in glycemic control and appetite, these hormones also exert skeletal effects. GLP-1 and GLP-2, produced in the ileum, suppress osteoclast-mediated bone resorption via their receptors (85). GLP-2 has been linked to increased bone density, and clinical studies indicate that GLP-2 analogs (e.g., teduglutide) can improve bone mass in patients with intestinal disorders. GIP similarly inhibits bone resorption and uniquely stimulates osteoblast activity and bone formation. By contrast, PYY—an anorexigenic gut hormone—inhibits osteoblasts and promotes osteoclast activity; clinically, elevated PYY levels are associated with lower bone mass. Overall, GLP-1/GLP-2/GIP tend to protect bone (by reducing resorption, with GIP also enhancing formation), whereas PYY may accelerate bone loss (85). These incretin pathways present potential therapeutic targets for OP.

#### 4.3.2 Microbiota regulation of incretin signaling

The gut microbiota modulates incretin secretion and metabolism via multiple mechanisms. SCFAs are important mediators: beyond their direct skeletal effects, SCFAs bind free fatty acid receptors (e.g., FFAR2) on L cells to induce GLP-1 and PYY release (86–88). High-fiber diets or prebiotic supplementation can increase SCFA production, thereby elevating GLP-1 levels and indirectly enhancing bone formation. The microbiota also influences L cell number and function; in models of type 2 diabetes, altering the microbiota restores GLP-1 secretion, implying that similar mechanisms may operate in OP (86). Additionally, microbially derived bile acids can stimulate gut hormone release via receptor-mediated pathways. Conversely, dysbiosis and intestinal inflammation can disrupt the incretin axis—for example, lowering GLP-2, which weakens intestinal calcium/phosphate absorption and diminishes gut support of osteogenesis (86). Restoring eubiosis can normalize GLP-2 and other hormone levels and thereby normalize bone turnover. Interestingly, GLP-1 receptor agonists (e.g., liraglutide, used in diabetes) have been associated with reduced fracture risk, potentially due to their anti-inflammatory effects and ability to decrease bone resorption (85). In summary, the gut microbiota influences bone homeostasis partly through incretins: microbial metabolites that boost GLP-1/GLP-2/GIP signaling (or reduce excess PYY) tend to decrease bone resorption and increase bone formation (85, 86). Elucidating this microbiota–incretin–bone axis may open new treatment avenues (e.g., “osteobiotics” or incretin-based therapies for OP).

### 4.4 Parathyroid hormone and the gut microbiota

#### 4.4.1 Microbial contributions to PTH-induced bone resorption

PTH is essential for calcium homeostasis and has biphasic effects on bone: chronically elevated PTH (e.g., in hyperparathyroidism) increases bone resorption and can cause OP, whereas intermittent low-dose PTH (teriparatide therapy) is anabolic. Recent studies suggest the gut microbiota is a critical “control valve” determining PTH’s bone effects (17, 89). In mice, Pacifici et al. found that depleting the gut microbiota with antibiotics prevented continuous PTH infusion from inducing bone loss; similarly, germ-free mice were resistant to PTH-induced bone resorption (17). These results imply that microbiota-dependent immune activation is required to fully drive PTH-mediated osteoclastogenesis. Mechanistically, specific taxa like SFB appear to mediate PTH’s catabolic effects: SFB promotes TNF-α^+^ T cells and Th17 cells in the gut, which migrate to bone marrow and secrete TNF-α and IL-17A. These cytokines amplify PTH-induced RANKL expression in bone marrow stromal cells, leading to vigorous osteoclastogenesis (17, 90). Likewise, Yu et al. reported that SFB-colonized mice given continuous PTH had more gut and bone marrow inflammatory cells and greater bone loss, whereas mice lacking SFB had significantly less bone loss (17, 91). Thus, pro-inflammatory gut microbes predispose the host to enhanced PTH-driven bone resorption (19, 70), potentially explaining inter-individual differences in hyperparathyroid bone disease and suggesting that combined microbiota-targeted interventions might be beneficial.

#### 4.4.2 Microbial support of intermittent PTH anabolism

Conversely, the microbiota also facilitates the anabolic effects of intermittent PTH. Li et al. showed that germ-free or antibiotic-treated mice had blunted responses to daily PTH injections, with less new bone formation and smaller gains in bone mass (28, 92). This defect appears to reflect a lack of butyrate: without commensals, systemic butyrate levels are low, and butyrate is permissive for PTH’s bone-building actions (92, 93). Butyrate stimulates Treg cells (via dendritic cells), and these Treg cells induce bone marrow CD8^+^ T cells to produce Wnt10b, activating Wnt signaling and osteogenesis (92, 94). In germ-free or dysbiotic mice lacking butyrate, this Treg–Wnt pathway fails to activate, impairing PTH-induced bone formation (92, 94). Notably, providing physiological doses of butyrate to germ-free mice partially rescued the bone anabolic response to PTH (92), indicating that microbial metabolites create a permissive milieu for PTH’s bone effects (95). This insight suggests a therapeutic strategy: co-administering PTH with prebiotics/probiotics that boost butyrate production might enhance anabolic efficacy. Overall, there is bidirectional crosstalk between PTH and the gut microbiota. Through immune and metabolic mechanisms, the microbiota can dictate the direction and magnitude of PTH’s skeletal effects—permitting or amplifying bone resorption with continuous PTH, and enabling bone formation with intermittent PTH (17, 92). Future microbiota-based interventions may allow fine-tuning of PTH’s actions while minimizing side effects.

## 5 Frontiers, challenges, and future directions

Despite mounting evidence implicating gut microbiota–immune–endocrine interactions in OP, research in this area is still in its infancy and faces many challenges (15). The gut–bone axis involves multi-layered, complex pathways, and the effects of specific microbial metabolites or immune mediators can be context-dependent (96). For example, butyrate stimulates osteogenesis at physiological concentrations but may inhibit cell growth at very high doses; low-dose LPS can induce immune tolerance, whereas high-dose LPS triggers severe inflammation and bone resorption (97). Likewise, a probiotic that improves bone outcomes in one model may be ineffective in another (98). Such context dependencies contribute to inconsistent results across studies and underscore the need for more precise mechanistic investigations. Further research should delineate strain-specific and metabolite-specific effects on bone under defined conditions, determine thresholds between beneficial and deleterious effects, and identify optimal intervention regimens (40).

Current knowledge is derived mostly from animal studies; relatively few clinical studies have been conducted, and some show conflicting results (96, 99). The human gut microbiome is highly complex and variable (influenced by geography, diet, genetics, etc.), resulting in significant individual heterogeneity (100, 105). Accordingly, small clinical trials often lack generalizability. To facilitate clinical translation, associations between specific gut microbial signatures and OP onset/progression should be validated in large-scale, longitudinal studies. Additionally, the efficacy and safety of microbiota-targeted interventions (e.g., probiotics and prebiotics) should be confirmed by large, rigorous randomized controlled trials (98, 101, 102). Currently, studies on probiotics in OP differ widely in strain selection, dose, and duration, which likely contributes to inconsistent outcomes. Standardized research guidelines and reporting frameworks (e.g., STORMS for microbiome studies) are needed to improve comparability and data quality across studies.

For more aggressive approaches like FMT in severe dysbiosis, safety in older patients with OP remains uncertain. FMT carries risks of infection and bacteremia, and immunosenescence plus co-morbidities in the elderly could heighten these risks. Careful patient selection and monitoring will be required if such interventions are attempted.

In short, the gut microbiota–immune–endocrine interactive system offers a paradigm shift in our understanding of OP (3). Recent evidence suggests that dysbiosis can promote bone loss by exacerbating inflammation, compromising the gut barrier, and disrupting hormonal signals; conversely, preventing or correcting dysbiosis—and restoring a healthy microbiota—may help defend the musculoskeletal system (103). Going forward, deeper mechanistic insights are needed, particularly into individual microbes and metabolites and their dose-dependent effects on bone, while maintaining focus on clinical validation and personalized approaches. Integrating multi-omics data and artificial intelligence can help map the gut–bone molecular network in greater detail, potentially revealing novel therapeutic targets. Looking ahead, modulation of the gut microbiota may complement conventional anti-osteoporotic therapies, providing synergistic benefits to the hundreds of millions affected worldwide. As accumulating evidence suggests, gut health and bone health are inseparable, and the gut microbiota may well become a critical “second battlefield” in OP prevention and treatment (14, 104).
